# Pathogenesis and novel therapeutics of regulatory T cell subsets and interleukin-2 therapy in systemic lupus erythematosus

**DOI:** 10.3389/fimmu.2023.1230264

**Published:** 2023-09-12

**Authors:** Yi-Giien Tsai, Pei-Fen Liao, Kai-Hung Hsiao, Hung-Ming Wu, Ching-Yuang Lin, Kuender D. Yang

**Affiliations:** ^1^Department of Pediatrics, Changhua Christian Children’s Hospital, Changhua, Taiwan; ^2^School of Medicine, Kaohsiung Medical University, Kaohsiung, Taiwan; ^3^School of Medicine, Chung Shan Medical University, Taichung, Taiwan; ^4^Department of Post-Baccalaureate Medicine, College of Medicine, National Chung Hsing University, Taichung, Taiwan; ^5^Division of Allergy, Asthma and Rheumatology, Department of Pediatrics, Chung Shan Medical University Hospital, Taichung, Taiwan; ^6^Department of Allergy, Immunology and Rheumatology, Changhua Christian Hospital, Changhua, Taiwan; ^7^Department of Neurology, Changhua Christian Hospital, Changhua, Taiwan; ^8^Division of Pediatric Nephrology, Children’s Hospital, China Medical University Hospital, Taichung, Taiwan; ^9^Department of Pediatrics, Mackay Memorial Hospital, New Taipei City, Taiwan; ^10^Institute of Clinical Medicine, National Yang Ming Chiao Tung University, Taipei, Taiwan

**Keywords:** systemic lupus erythematosus, lupus nephritis, regulatory T cells, interleukin-2, B regulatory cells

## Abstract

Systemic lupus erythematosus (SLE) is a heterogeneous multisystem inflammatory disease with wide variability in clinical manifestations. Natural arising CD4+ regulatory T cells (Tregs) play a critical role in maintaining peripheral tolerance by suppressing inflammation and preventing autoimmune responses in SLE. Additionally, CD8+ regulatory T cells, type 1 regulatory T cells (Tr1), and B regulatory cells also have a less well-defined role in the pathogenesis of SLE. Elucidation of the roles of various Treg subsets dedicated to immune homeostasis will provide a novel therapeutic approach that governs immune tolerance for the remission of active lupus. Diminished interleukin (IL)-2 production is associated with a depleted Treg cell population, and its reversibility by IL-2 therapy provides important reasons for the treatment of lupus. This review focuses on the pathogenesis and new therapeutics of human Treg subsets and low-dose IL-2 therapy in clinical benefits with SLE.

## 1 Introduction

**Figure 1:**
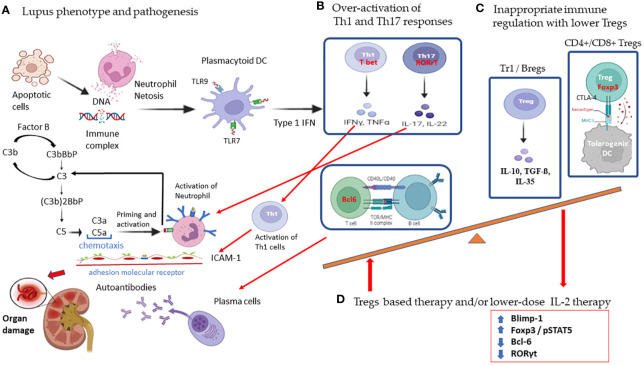
Schematic diagram showing the pathogenic mechanism of overactivated T cell responses and the therapeutic potential of regulatory cells with low-dose IL-2 therapy in systemic lupus erythematosus. **(A)** SLE is characterized by increased apoptosis and defective clearance mechanisms leading to the formation of immune complexes against the self-antigen. These immune complexes cause neutrophil netosis and activate plasmacytoid dendritic cells to interact with self-reactive pathogenic T cells. **(B)** Overactivation of Th1 and Th17 responses results in high expression of type 1 interferon, cytokines, and adhesion molecules that increase tissue damage and induction of anti-DNA autoantibodies. **(C)** Subsets of skewed lower Treg cells with inappropriate immune homeostasis will contribute to the worsening of lupus. **(D)** IL-2 induces Tregs differentiation in association with STAT5 phosphorylation with sustained expression of FoxP3. IL-2 directly inhibits expression of RORγt and blocks Th17 development. IL-2 also induces Blimp-1 to decrease Bcl-6 expression in T follicular helper cells. Low dose IL-2 therapy restores Treg suppressive function mainly by secreting inhibitory cytokines with IL-10, TGF-β, IL-35, and cytolysis with granzyme B and perforin, and deprivation of effector T cell growth with IL-2 and suppression of dendritic cell function. (created with BioRender.com). Agreement number: QI25RDVKPZ.

Systemic lupus erythematosus (SLE) is a chronic multisystem systemic inflammatory disorder characterized by an overactivated innate immune response, uncontrolled autoantibody production, and the presence of self-antigens that induce the deposition of immune complexes (1, 2). Endogenous self-antigen and nucleosomes from apoptotic cells in SLE will activate plasmacytoid dendritic cells (DCs) through the Toll-like receptor (TLR) 7 and TLR 9 to interact with pathogenic self-reactive T cells for type 1 interferon production, and with B cells for the manufacture of autoantibodies (2–5). The inappropriate stimulation of Th1 and Th17 inflammatory cytokines and the loss of self-tolerance will contribute to immune dysfunction and tissue inflammation in SLE (1, 6, 7). As shown in **Figure 1**, we summarize the pathogenesis of SLE in the overactivation of Th1 and Th17 responses with lower regulatory T cells (Tregs) resulting in a high expression of type 1 interferon cytokines and adhesion molecules that increase neutrophil netosis and tissue damage and apoptosis with nuclear DNA exposure for the induction of anti-DNA autoantibodies. Skewed lower Treg cells attributed to inappropriate immune homeostasis and proper targeting of imbalanced Treg function and/or low-dose IL-2 the administration could rescue the progress of SLE.

Tregs are vital for maintaining immune self-tolerance and regulation of immune tolerance by inhibiting autoimmune and inflammatory responses (8–11). Natural CD4+ Tregs (nTregs) are derived from thymic precursors, consisting of approximately 5% to 10% of the circulating population of CD4+ T cells (12, 13). The nTregs had high expression of CD25 and low expression of CD127 in CD4+ T cells, and the transcription factor forkhead box P3 (FoxP3) served as the best known Treg hallmarks (12, 14, 15). It is important to note that genetic factors contribute to the risk of developing SLE and immune dysregulation (16). Certain genes involved in T-cell activation, regulation, and type I interferons have been identified as risk factors for SLE susceptibility (17). For example, variations in the FoxP3 gene, which encodes the critical transcription factor for Treg development and function, have been linked to impaired Treg function in SLE patients. FoxP3 induces the expression of IL-2, CD25, CTLA-4, and miR-155 in Tregs (18). GDF7, a secreted ligand of the TGF-β superfamily of proteins, exhibited positive regulatory effects on Tregs via increasing the expression of FoxP3 and CTLA4. Recent research showed that the downregulation of GDF7 in CD4+ T cells could lead to impaired suppressive functions of lupus Tregs (17, 19). Natural CD4+ Tregs are rendered unstable by loss of FoxP3 expression, which can ultimately lead to autoimmunity. FoxP3 mutation has been reported as a rare X-linked recessive (IPEX) syndrome with aggressive autoimmunity with the clinical feature of immune dysregulation, polyendocrinopathy, and enteropathy in humans (9, 20). Epigenetic modifications, which can be influenced by both genetic and environmental factors, also impact T-cell function in SLE. The interaction between genetics, T cell abnormalities, and environmental factors also plays a critical role in in the onset and exacerbation of the disease (16).

Natural Tregs-mediated immune regulation is mediated primarily by cell contact and suppressive cytokines (**Table 1A**). The immunoregulatory mechanism of nTregs includes the secretion of inhibitory cytokines with IL-10, IL35, TGF-β, and cell-contact inhibition with perforin and granzyme B, and the competition of IL-2 with effector T cell growth, and suppression of DC function (8). The tolerance of DCs can be induced by Tregs through positive feedback loops to reinforce the suppressive environment, and CTLA-4 expressed by Tregs can inhibit the expression of CD80 and CD86 on DCs to down-regulate effector T cells via the costimulatory cell surface protein CD28. Tolerogenic DCs can express inhibitory molecules such as indoleamine 2,3-dioxygenase (IDO) and retinoic acid, to improve Treg survival and proliferation to suppress autoimmunity (21).

**Table 1:** The immune mechanism of human regulatory T cell subsets and regulatory B cells.

Subtype		Origin	Markers/(transcription)	Induction factor	Major suppressive mechanism
**(A)**	CD4+Tregs	Thymus	CD4^+^, CD25^+^, CD127^-^, CTLA-4^+^, GITR^+^, CCR6^+^, CD45RA^+^, CD45RO^+^, (**FoxP3^+^**)	TCR/CD28 affinity-dependent IL-2	(1) Secreting inhibitory cytokines, including IL-10, TGF-β, IL-35. (2) Cell contact, killing effector cells by granzyme and perforin. (3) Metabolic competition, deprivation the growth of effector T cells requires the maintenance of IL-2. (4) Suppression of dendritic cell maturation and function.
**(B)**	CD8+ Tregs	Peripheral	CD8^+^, CD25^+^, CD39^+^, CD26^-^, CXCR3^+^, ICOS^+^, CTLA-4^+^, CD103^+^, CD28^+^, CD122^+^, 41BB^+^, LAG^+^, (**FoxP3^+^**)	Antigen-presenting cell (APC) and TGF-β and IL-2	(1) Direct death of effector cells by granzyme and perforin. (2) Negative signaling through CTLA-4 to suppress APC. (3) Secreting inhibitory cytokines, including IL-10, TGF-β, IL-35. (4) Metabolic competition, deprivation the growth of effector T cells requires the maintenance of IL-2.
**(C)**	Bregs	Peripheral	BCR^+^, MHC-II, CD40^+^, CD19^+^, CD1d^hi^, CD5^+^, CD9^+^, CD25^+^, CD27^+^, CD24^hi^, CD38^hi^, CD71^hi^, CD73^lo^, TLRs^+^, (**AhR**)	BCR, CD40- CD40L signaling, IL-21, TLR signaling	(1) Secreting inhibitory cytokines, including IL-10, TGF-β, IL-35.
**(D)**	Tr1	Peripheral	CD4^+^, TCR^+,^ LAG3^+,^ CTLA-4^+^, PD-1^+^, CD49b^+^, CCR5^+^, (**Blimp**, **AhR**)	Tolerogenic dendritic cells, IL-10, TGF-ß	(1) Secretion of IL-10, IL-35, TGF-ß, perforin, granzyme B.
**(E)**	CD46-Tr1	Peripheral	CD4^+^, CD46^+^, (**Cyt1, Cyt2**)	Anti-CD3, Anti-CD46, IL-2	(1) Secretion of IL-10, IFN-γ, TGF-ß, granzyme B.

## 2 Imbalance of immune regulatory cells in SLE

Numerical and functional deficits of abnormalities in Treg subtypes have been reported to have a significant impact on the pathogenesis and outcome from patients with autoimmune disorders (22–26). Elucidation of the roles of various subsets of Treg (**Table 1**) dedicated to immune balance will provide a novel therapeutic approach that governs immune tolerance for SLE remission (27, 28). However, no definitive phenotypic markers or functions have been recognized to distinguish between these Treg subtype populations in SLE. In this mini-review, we summarize the current literature on the function and diversity of Treg subsets, focusing mainly on CD4+ Tregs, CD8+ Tregs, B regulatory cells, and type 1 regulatory T cells (Tr1), which provide immunotherapeutic modulation of SLE (**Table 2**). Furthermore, we discuss the clinical trials and therapeutic potential of regulatory cell-based therapies and lower-dose IL-2 therapy to restore immune tolerance by Tregs manipulation in patients with SLE.

**Table 2:** Characteristics and main findings of regulatory T cell subsets and regulatory B cells in systemic lupus erythematosus.

Treg cell subsets		Study Design/ Animal models	Sample Size/Species	Main finding	References
**(A)**	CD4+ Tregs	Meta-Analysis	1431 SLE patients and 1056 health controls.	SLE patients defined by CD4+CD25+FoxP3+, CD4+CD25+, and CD4+FoxP3+ had a much lower Treg/PBMC ratio compared to healthy controls.	(29)
	CD4+ Tregs	Meta-Analysis	628 active SLE patients and 601 health controls.	The pooled absolute numbers of Tregs in active SLE decreased significantly compared to controls and the results were not affected by gating strategies.	(30)
	CD4+ Tregs	Meta-Analysis	1772 active SLE patients and 1007 health controls.	The proportion of Tregs/CD4+Tcells was much lower in patients with active SLE compared to those with inactive SLE.	(31)
	CD4+ Tregs	Case control	47 patients with SLE and 15 healthy donors.	SLE patients had circulating high Th17 and lower Tregs with decreased miR-19b expression.	(32)
	CD4+ Tregs	Murine model	lupus MRL/lpr	Treg cells reduced suppressive capacity against T cell proliferation.	(33)
	CD4+ Tregs	Murine model	NZB × NZW F1 lupus mice	Adoptive transfer of Treg cells leads to disease suppression and remission. Administration of recombinant recombinant IL-2 could restore the imbalance of effector T cells and proliferation of Treg cells.	(34, 35)
**(B)**	CD4+ Follicular Tregs	Case control	19 patients with SLE and 14 healthy donors.	Tfr cells from patients with SLE had impaired suppressive function. PD-1+ Tfr cells in SLE positively correlated with anti-DNA antibody levels and disease activity.	(36)
	CD4+ Follicular Tregs	Case control	41 patients with SLE and 26 healthy donors.	Impaired CD4+CXCR5+CD45RA-FoxP3^high^ Tfr cells could be attributed to defective IL-2 production in patients with SLE.	(37)
	CD4+ Follicular Tregs	Case control	58 patients with SLE and 24 healthy donors.	An increase in circulating Tfrs numbers was observed with successful treatments in patients with SLE.	(38)
	CD4+ Follicular Tregs	Murine model	lupus MRL/lpr	The percentage of Tfh cells increased and the Tfr (CD4+CXCR5+FoxP3+) cells decreased in 16-week-old MRL/lpr mice.	(39)
**(C)**	CD8+ Tregs	Case control	40 patients with Class III/IV lupus nephritis and 10 healthy controls.	Patients with SLE treated with methylprednisolone have CD8+CD25+FoxP3+Tregs associated with decreased disease activity.	(40)
	CD8+ Tregs	Case control	30 patients with SLE and 15 healthy controls.	A nucleosomal histone peptide induces CD8+CD25+FoxP3+ T cells suppressed pathogenic autoantibody production by TGF-β/ALK-5/pSmad 2/3 signaling.	(41)
	CD8+ Tregs	Case control	15 post-transplant, 10 drug-treated patients with SLE and 15 controls.	Autologous hemopoietic stem cell transplantation restores CD8+FoxP3+ Treg cells in SLE.	(42)
	CD8+ Tregs	Chronic GVHD lupus nephritis murine model	B6D2F1 mice	Adaptive transfer of suppressive CD8+CD103+CD39+ Tregs inhibited chronic graft versus host disease in murine lupus nephritis model.	(43)
	CD8+ Tregs	Murine model	NZB × NZW F1 lupus mice	Plasmid DNA vectors encoding epitopes activated CD8+ cytotoxic T cells to kill autoantibody-producing B cells and ameliorated the severity of lupus. Suppressive properties of CD8+ Tregs could be manipulated by administering pConsensus peptides to suppress lupus nephritis with anti-dsDNA autoantibodies.	(44–46)
**(D)**	Bregs	Case control	41 patients with SLE and 25 healthy controls	CD19^+^CD24^high^CD27^+^B population with IL-35 decreased in active SLE patients, and correlated with disease activity.	(47)
	Bregs	Case series	25 new-onset lupus nephritis patients.	CD19^+^CD5^+^CD1d^high^IL-10^+^ Bregs were deficient in new-onset lupus nephritis patients and increased in responders with immunosuppression	(48)
	Bregs	Case control	47 newly diagnosed SLE and 20 healthy controls.	SLE patients had a decrease in IL-35+ B cells and IL-10+ B cells and plasma IL-35.	(49)
	Bregs	Case control	116 patients with SLE and 88 healthy controls.	Bregs limit the production of IFN-r by pDCs through an IL-10-dependent mechanism. In SLE patients receiving Rituximab antibody, the relationship between pDCs and Bregs is normal.	(50)
	Bregs	Case control	34 patients with SLE and 21 healthy controls.	Circulating CD19+CD24^hi^CD38^high^ cells were not different between SLE patients and controls. The percentages of IL-10+ Bregs were significantly decreased in lupus nephritis.	(51)
	Bregs	Case control	18 patients with SLE and 13 healthy controls.	The capacity of B cells from lupus patients to produce IL-10 cytokines upon TLR9 engagement becomes less efficient with increasing disease activity.	(5)
	Bregs	Case control	55 new-onset lupus patients and 36 controls.	Circulating CD24^high^CD27+ CD19+ B cells was significantly reduced in patients with SLE, and correlated SLEDAI scores.	(52)
	Bregs	Case control	24 active lupus patients and 14 healthy controls.	CD40 stimulates CD19+CD24^high^CD38^high^B cells to suppress Th1 differentiation via IL-10. SLE patients were refractory to CD40 stimulation and produced less IL-10.	(53)
	Bregs	Murine model	MRL/lpr mice	Tfh cell-derived IL-21 drove the differentiation and IL-10 production of B10 cells.	(54)
	Bregs	Murine model	Roquin^san/san^ mice	The deficiency of IL-17 led to an increased number of CD19+IL-10+Breg in the spleen of a murine model of lupus.	(55)
	Bregs	Murine model	MRL/lpr mice	DNA of gut microbiota has been shown to expand the Bregs.	(56)
**(E)**	Tr1	Case control	10 lupus patients and 6 healthy controls.	Decreased ability to limit autoantibody production by B cells.	(57)
**(F)**	CD46-Tr1	Case control	40 patients with active Class III or IV LN and 30 healthy controls.	Intravenous methylprednisolone therapy restored CD3/CD46-activated Tr1 cells with IL-10 and alter CD46-Cyt1/Cyt2 patterns.	(58)
	CD46-Tr1	Case control	45 patients with SLE and 38 healthy controls.	CD46-regulated Th1 contraction (IFN-γ to IL-10 switching) is defective in Th1 cells from patients with SLE. MMP-9 increases the shedding of soluble CD46 by Th1 cells associate with this defect.	(59)
	CD46-Tr1	Case control	152 patients with SLE and 80 healthy controls.	lupus nTreg dysfunction is not due to intrinsic defect, but is induced by C3b stimulation of CD46 and IFN-α and these immune components of inflammation are directly associated with active lupus.	(60)

### 2.1 Natural regulatory T cells in SLE

Controversial results were reported whether decreased and increased Tregs cells frequencies were reported in SLE. This discrepancy may arise from the difference in phenotypic analyses and the composition of study cohorts. Control groups in clinical trials are made up of patients who receive a standardized treatment or placebo. The baseline characteristics and ongoing treatments of these patients can affect Treg populations. For example, high-dose glucocorticoid therapy has been found to increase the percentage of circulating Tregs in active lupus patients (61). Although circulating CD4+Treg levels have concluded inconsistent results, there is increasing evidence to specify that the numbers of CD4+Tregs were substantially reduced in patients with lupus (**Table 2A**). A meta-analysis showed that a lower percentage of Tregs/CD4+T cells was observed in patients with active lupus compared to those with inactive SLE (31). A recent meta-analysis showed that SLE patients had a decreased Treg/PBMC ratio, which Treg is defined by CD4+CD25+, CD4+FoxP3+ and CD4+CD25+FoxP3+ compared to healthy subjects. However, no significant differences were found between the percentage of CD4+Tregs and the activity of SLE disease due to the sample size or inclusion criteria (29). Another meta-analysis showed that patients with active SLE have a decreased absolute number of CD4+Tregs and the results were not affected by the gating strategies (30). However, considering that most patients with lupus received glucocorticoid, CD4+Treg has been reported to be unaffected by drug therapy (62). Furthermore, lupus patients have an increased rate of CD4+ Tregs apoptosis and a decrease in total Tregs count, and CD4+Tregs apoptosis which was positively associated with lupus disease activity (63).

Th17 cells play a critical role in the pathogenesis of SLE and several evidences have confirmed that refining the balance of CD4+Treg/Th17 cells could benefit from reducing disease activity (32, 64). The disturbance between Th17 and CD4+Treg proliferation was highlighted by an imbalance in FoxP3 and RORγt gene expression in the development of SLE (6). Dysregulated Th17/CD4+ Treg cell balance was associated with miR-19b gene expression. Exosomes from human umbilical cord blood-derived mesenchymal stem cells could regulate the expression of inflammatory factors through *in vitro* miR-19b/KLF13 experiments in patients with lupus (32) (**Table 2A**). In lupus murine models, CD4+Foxp3+ Treg cells reduced the suppressive capacity against T cell proliferation, and adoptive transfer of Treg cells leads to lupus disease suppression and remission (33). Administration of recombinant IL-2 could restore the imbalance of effector T cells and proliferation of Treg cells (34, 35) (**Table 2A**).

The T follicular helper cells (TFh), which are defined by a phenotype of Bcl6+CXCR5^high^PD-1^high^, in the germinal center had the ability to stimulate the follicular B cells for the production of high-affinity autoantibodies. Several studies discovered an increase in the number of peripheral TFh cells in lupus patients, which is positively correlated with disease activity and autoantibody titers (65). Circulating lymphoid tissue follicular Treg cells (TFr), which expressed PD-1, ICOS, CD25 and CXCR5, can suppress TFh cell activity by inhibiting IL-10, IL-35, and TGF-β cytokine production (66). An increase in circulating TFr cell numbers was observed with successful treatments in patients with SLE, suggesting that restoring TFr cells may provide new therapeutic methods in SLE (38) (**Table 2B**). In lupus murine models, the percentage of Tfh cells increased and the Tfr (CD4+CXCR5+FoxP3+) cells decreased in 16-week-old MRL/lpr mice (39) (**Table 2B**).

The disruption of CD4+Treg cell homeostasis triggered by the lack of IL-2 is a critical event in the pathogenesis of SLE (67). The number and function of CD4+Treg can be controlled by the IL-2/STAT5 signaling pathway with sustained expression of FoxP3. IL-2 has also been reported to increase TFr proliferation which is dependent on the STAT5/Blimp-1 dependent mechanism (68). Impaired CD4+CXCR5+CD45RA-FoxP3^high^ TFr cells with defective IL-2 production could be found in patients with SLE (69). Furthermore, T cells from SLE patients exhibit abnormal microRNAs (for example, miR-200a-3p) and enzymatic activity of protein phosphatase 2A (PP2A), resulting in downregulation of IL-2 by inactivation of the phosphorylated cAMP response element-binding protein (pCREB) (16, 70).

### 2.2 CD8+ regulatory T cells in SLE

Cytotoxic Tregs are heterogeneous in periphery circulation and retain the ability to destroy T cell activation and proliferation to ensure immune homeostasis (71). CD8+ FoxP3+ Treg cells were first recognized in human tonsils, and CD8+ Treg cell subsets based on CD25+ expression may share similar suppressive capacity typically associated with CD4+ Treg (28, 71, 72). Isolated human CD8+ Tregs frequently express several cell surface molecules that include CD8, CD103, CD25, CD39, CD28, ICOS, CD122, CD39, CTLA-4, CXCR3, 4-1BB and lymphocyte activation gene 3 (LAG-3) (73). Suppressive CD8+FoxP3+ Treg cells could decrease the proliferation of CD4+ effector T cells through the above mechanism, including cell-cell contact lysis through the granzyme/perforin, Fas/FasL signaling pathways and secreting inhibitory cytokines, and deprivation effector T cell growth by metabolic competition of IL-2 (28, 71, 74–76) (**Table 1B**).

Loss of homeostatic balance is observed in lupus patients and restored human CD8+ Treg cells can be vital for the development of therapies in SLE (77). A decrease in circulating CD8+ FoxP3+ Treg cells has been observed in SLE patients, and autologous hemopoietic stem cell transplantation could increase CD8+Treg cells, which are related to better control (42). High-dose methylprednisolone therapy could increase circulating CD8+CD25+FoxP3+Tregs and decrease disease activity in patients with lupus nephritis (40). All-trans retinoic acid has been shown to increase the CD4+ and CD8+ regulatory T Cells, which have therapeutic potential in the treatment of SLE and other autoimmune diseases (78) Furthermore, a nucleosomal histone peptide could improve human CD8+CD25+FoxP3+ T cells to decrease the production of pathogenic autoantibody via TGF-β/ALK-5/pSmad 2/3 signaling pathway (41) (**Table 2C**).

Defective CD8+ Treg cells and function were found in murine lupus nephritis models (28), and adaptive transfer of suppressive CD8+CD103+CD39+ Tregs could inhibit chronic graft versus host disease in murine lupus nephritis model (43). Transfection plasmid DNA vectors encoding epitopes activated CD8+ cytotoxic T lymphocytes (CTL) to kill autoantibody-producing B cells and ameliorated the severity of lupus (44). Immune tolerance induced by anti-DNA-based peptides (pConsensus, pCons peptide) could improve human CD8+ Tregs to decrease proliferation of naïve CD4+ T cells and B cells (73). The suppressive properties of CD8+ Tregs could be manipulated by administering pCons peptides to suppress lupus nephritis with anti-dsDNA autoantibodies in the lupus murine model (NZB x NZW F1) (45, 46) (**Table 2C**).

### 2.3 Regulatory B cells in SLE

SLE is associated with abnormal activation of B lymphocytes and excessive autoantibodies production. Regulatory B (Breg) cells have an important role in the maintenance of immune tolerance and attenuating systemic inflammation (47). Human Bregs are generally recognized to have a phenotype of CD24^high^CD27+ or CD19+CD24^high^CD38^high^ (52, 53). Human B10 cells, which constitute approximately 25% of B cells in the blood, are activated by the B-cell receptor (BCR) of progenitor B10 cells (79, 80). B10 cells as a functional Bregs subtype have a negative immunoregulatory function, and CD40 and CD40L signaling, apoptotic cells, and TLR signaling were able to increase the number of B10 cells (53, 80, 81). pDCs can induce Breg differentiation via an IFN-r-dependent pattern by secreting IL-10 and IL-35 (49, 51) (**Table 1C**).

Emerging evidence has revealed that Breg numerical deficiency and/or dysfunction play a critical role in the pathogenesis of lupus and other autoimmune diseases (47, 82, 83). Identification and characterization of Bregs facilitate new approaches to targeting pathogenic B cells in patients with SLE (84) (**Table 2D**). Impaired regulatory B cells producing granzyme B were found in patients with SLE (82). Patients with lupus nephritis had a significantly decreased in circulating IL-10+ Bregs levels compared to health controls (51). Patients with new-onset lupus nephritis had a decrease in CD19+CD5+CD1d^high^IL-10+ Bregs and restored Breg deficiency in patients who respond to immunosuppressive medications (48). The percentage of peripheral CD19+CD24^high^CD27+ B cells with IL-10 and IL-35 production was low in patients with new-onset SLE (47, 52). Granzyme B-producing Bregs, which play a negative regulatory role in immunity, and Granzyme B-producing B cell frequencies decreased in patients with SLE (82). During CD40 stimulation, decreased CD19+CD24^high^CD38^high^ B cells with IL-10 production and inhibitory capacity was observed in SLE patients (85). pDCs initiative the differentiation of CD19+CD24^high^ CD38^high^ (immature) B cells into IL-10-producing CD24+CD38^high^ Breg cells. Defective pDC-mediated expansion of CD24+CD38^high^ Breg cells was associated with decreased activation of the STAT1 and STAT3 signaling pathway in patients with SLE. STAT1-STAT3 activation between pDC and CD24+CD38^high^ Breg cell interaction was restored in SLE patients responding to rituximab, a chimeric monoclonal antibody targeted against the CD20 marker (50).

Bregs and T cells can influence each other on the functions through direct cell-cell interactions and the production of cytokines. Dysfunctional Bregs may provide inadequate regulatory signals to T cells, leading to unchecked pro-inflammatory responses. Bregs exert profound impacts on the differentiation, function, and distribution of Tfh cells in the immune microenvironment. Human Breg cells control IL-21 receptor expressions in Tfh cell maturation, and inhibit Tfh cell–mediated antibody secretion through IL-10 and TGF-β (86). Human CD19+CD25^high^ Bregs can inhibit the expansion and function of autologous CD4+ T cells, and promote Treg differentiation and activity by secreting IL-10 (87). The IL-10-independent regulatory mechanisms of Bregs to inhibit effector T cell differentiation are primarily mediated by IL-35, TGF-β, and granzyme B (88). Programmed death-ligand 1 high (PD-L1^hi^) Bregs inhibit Tfh cell expansion through elevated expression of PD-L1 (89). Furthermore, specific IgG4 antibodies produced by human CD73−CD25+CD71+IL-10-producing regulatory B cells could suppress antigen-specific CD4+ T cell proliferation (90). Enhancing Breg function or adoptive transfer of Bregs in animal models of SLE has shown promising results in reducing disease severity and ameliorating T-cell abnormalities. The development and function of Bregs are likely to be regulated by Tfh cells, and the percentage of B10 cells in the spleens had a positive correlation with Tfh cell-derived IL-21 from a MRL/lpr mice model (54). IL-17 deficiency led to an increased number of CD19+IL-10+Breg in the spleen of a murine model of lupus (55). The gut microbiota DNA has been shown to expand the Bregs in a murine lupus model (56) (**Table 2D**). Collectively, all these data support the fact that Breg cells are critical in T cells homoeostasis to ensure central tolerance and prevent autoantibodies production, and may have implications for the regulation of autoimmune diseases.

### 2.4 Tr1 regulatory cells in SLE

Periphery Tregs developed in conventional CD4+ T cells after stimulation with tolerogenic antigens are termed iTregs (58, 60, 91, 92). IL-10 producing T regulatory type 1 (Tr1) cells are induced in peripheral CD4+ T cells after antigen through the secretion of IL-10 and TGF-β (91, 92) (**Table 1D**). Tr1 cells secreting IL-10 are important in the regulation of immune tolerance to reduce tissue damage in SLE (58, 60). The aryl hydrocarbon receptor (AHR) and Blimp-1 are mandatory for Tr1 differentiation with IL-10 production (91, 93). IL-10R signals through the STAT3 and p38/MAPK signaling pathway will result in expansion of peripheral blood Tr1 cells (94). Furthermore, tolerogenic DCs producing IL-10 (DC-10) isolated from human peripheral blood are potent inducers of adaptive Tr1 cells through the IL-10–dependent ILT4/HLA-G signaling pathway (91, 95).

Tr1-like cells play a crucial role in suppressing antibody-driven immune responses. The function with inhibition of IgG production from Tr1-like cells was deficiency in SLE patients (57) (**Table 2E**). CD46, the complement regulatory protein, will be expressed in activated immune cells to prevent autologous complement-mediated lysis of cells by binding to complement components C3b and C4b in an inflammatory situation (59, 96). The uncontrolled immune response of Th1 contributes to a wide range of lupus, and CD46 activation has emerged as a powerful controller of T cell-mediated immunity to contraction of Th1 immunity (59). Activation of CD46 proteins with IL-2 in activated CD4+ T cells will enhance the secretion of IL-10 and granzyme B, a phenotype shared with Tr1 cells (60, 96, 97) (**Table 1E**). Costimulation by CD3/CD46 proteins leads to IL-2 dependent Tr1-like cells with high IL-10 and low IFN-γ level production, which is associated with disease activity in lupus patients (59, 98). Furthermore, we observed that the functional defect of IL-10+ Tr1-like cells in pediatric patients with lupus nephritis can be restored by corticosteroid treatment (58) (**Table 2F**).

## 3 Therapeutic potential of regulatory cells in SLE

### 3.1 Targeted-Treg therapies

Regulatory cell-based therapy can restore the homeostatic balance in the immune system (3, 99, 100). A variety of target regulatory cell-based therapies are currently being explored for SLE (**Table 3**). Conventional immunotherapy for SLE is mainly based on corticosteroids or/and immunosuppressive therapy (66, 77, 108). Restoration and maintenance of the Treg cell population have the therapeutic potential to improve immune tolerance by manipulating suppressive Tregs in patients with SLE (109–111). High-dose corticosteroid pulse therapy has been shown to induce monocytes to produce TGF-β and improve Treg differentiation. Furthermore, steroids play a role in activating Tregs through the miR-342-3p-mTOR complex 2 axis (22, 40, 101, 112) (**Table 3A**). Furthermore, antigen-specific Tregs can be triggered and expanded by administering autologous tolerogenic DCs with autoantigenic peptides and can be considered as a type of Treg therapies targeted against autoimmunity (22, 27) (**Table 3B**).

**Table 3:** Target regulatory cell-based therapies for systemic lupus erythematosus.

Regimens		Effects/Mechanism	References
**(A)**	Corticosteroid therapy	Methylprednisolone pulse therapy induces CD4+ T cell apoptosis, which promotes monocytes to produce TGF-β and further facilitates Treg differentiation. Steroids induce Tregs through miR-342-3p-mTOR complex 2 axis.	(22, 40, 101)
**(B)**	Tolerogenic dendritic cells	Tolerogenic DCs secrete anti-inflammatory cytokines, secrete retinoic acid, and indoleamine 2,3-dioxygenase (IDO), both of which enhance Treg survival and proliferation.	(22, 27)
**(C)**	Rapamycin/Retinoic acid	The mammalian target of the rapamycin inhibitor (mTOR) and retinoic acid expands Tregs and maintains suppressive function.	(102, 103)
**(D)**	Histone peptide tolerance	Histone peptide epitope and anti-DNA-based peptides (pConsensus) expansion CD4+ and CD8+ Tregs and suppress CD4+ T cells and B cell proliferation.	(41, 64, 73, 104)
**(E)**	Autoantigen-targeted Treg cells	Administering antigen-specific nanoparticles to Tregs and engineering the Tregs to be autoantigen-specific T cell receptor (TCR) or chimeric antigen receptor (CAR) Tregs can be directly inhibited in self-reactive cells. A nucleosomal histone peptide induces human CD8+CD25+FoxP3+ T cells to suppress pathogenic autoantibody production by TGF-β/ALK-5/pSmad 2/3 signaling.	(22, 105)
**(F)**	Low-dose IL-2 therapy	IL-2 increased STAT5 phosphorylation with sustained expression of FoxP3. IL-2 directly inhibits RORγt and induces Blimp-1 to decrease Bcl-6 expression.	(68, 106, 107)

Several studies have also investigated the mammalian target-of-rapamycin (mTOR) signaling pathway in Tregs and its impact on SLE pathogenesis (102). Rapamycin alone or in combination with all-trans retinoic acid reduced disease activity and the glucocorticoid requirement, which can regulate the balance of Th17/Treg cells (102, 103) (**Table 3C**). The tolerance of histone peptide was shown to be a novel therapy suitable for the treatment of lupus nephritis and restored Th17/Treg cell balance (64). Potent Treg cells are activated by histone peptide epitopes *in vitro* from patients with lupus, which can inhibit interferon and anti-dsDNA autoantibody production (41, 73, 104) (**Table 3D**). Treg therapy can be further enhanced by administering antigen-specific nanoparticles, or engineering Treg cells to be autoantigen-specific T cell receptor (TCR) or chimeric antigen receptor (CAR)-specific Tregs (22, 105) (**Table 3E**).

### 3.2 Low-dose IL-2 therapy on the balance of Tregs

CD25 is persistently expressed in CD4+ FoxP3+ Tregs, when IL-2 activated CD25 forming high affinity IL-2R to compete for available IL-2 in the circulation (113). The decrease in IL-2 level was found to be associated with a depleted Treg cell population, and its reversibility by IL-2 therapy provides important reasons for the treatment of lupus (114, 115). High doses administration of recombinant human IL-2 (rIL-2) can aid conventional T cells and naïve CD8+ T cells that produce perforin, granzymes, IL-5, IL-13, and IFN-γ (116). However, low-dose rIL-2 management preferentially promoted Treg cell-mediated beneficial activities in disease manifestations (105, 106).

The benefit of low-dose IL-2 therapy was associated with restored Tfr/Tfh cell balance. In CD4+ T cells, IL-2 can directly activate Blimp-1 and suppress Bcl-6 to antagonize Tfh cell differentiation (106). Furthermore, IL-2 therapy also directly prevents the expression of RORγt, thereby repressing Th17 development, which plays a vital role in the development of autoimmune disorders (107) (**Table 3F**). Tfr cells from SLE patients had reduced suppressive function. PD-1+ Tfr cells in SLE were positively correlated with disease activity and anti-DNA antibody levels. With *in vitro* low-dose IL-2 stimulation, the expression of PD-1 in Tfr cells decreased significantly, together with the increased expression of CTLA-4 and FoxP3 (36). Low-dose IL-2 therapy increases the circulating Tfr/Tfh ratio, which was accompanied by reduced anti-dsDNA titers and improved kidney damage in mice and lupus patients (117). A randomized cohort study showed that patients with lupus who received prolonged low-dose IL-2 therapy recoveed the immune balance Tfr/Tfh in lupus (65, 118).

### 3.3 Summary of IL-2 clinical trials for SLE

Humrich et al. (119) first reported that a 36-year-old patient with SLE who received human IL-2 recombinant (aldesleukin) 1.5 or 3.0 million IU subcutaneous injections daily for five days can improve arthritis and the increase of CD25+FoxP3+CD127^lo^ Treg cells. Clinical and preclinical studies demonstrate that low-dose rIL-2 can be administered safely to humans and the potential benefits in the induction of remission in active lupus patients as shown in **Table 4**. The low-dose recombinant human rIL-2 regimens contain 3-4 cycles of 7-10 million IU and are separated by rest periods of 9-16 days (37, 118, 120–124). Recent two randomized trials have shown that low-dose IL-2 therapy can restore the Treg population in active lupus patients. A randomized, double-blind, placebo-controlled clinical trial was designed to treat active lupus patients who received IL-2 at a dose of 1 million IU or placebo with standard treatment for 3 months and were followed for another 3 months. In the IL-2 group, the SRI-4 response rates were 55.17% compared to 30.00% in the control group. Although the SRI-4 response did not achieve a significant difference at 3 months, the IL-2 group had significantly high SRI-4 response rate after 6 months follow-up. In the IL-2 group, patients with lupus nephritis had a complete remission rate of 53.85% compared to 16.67% in the placebo group. No obvious systemic side effect was observed in the IL-2 group. Low-dose IL-2 therapy can expand regulatory T cells and also maintain cellular immunity with improved natural killer cells (118). An international, multicenter, double-blind, randomized, placebo-controlled phase II clinical trial (LUPIL-2) to enroll hundred lupus patients with moderate to severe activity. The patients were randomly assigned 1:1 to receive 1.5 million IU/day of subcutaneous IL-2 (ILT-101) or placebo for five days and followed by weekly injections of 3 months. In the intention-to-treat population, the SRI-4 response was not met (ILT-101: 68% *vs.* placebo: 58%; *P* =0.34), due to a 100% SRI-4 response rate at two sites in the control group. After excluded patients from these two sites, the SRI-4 response rate showed a statistically significant difference (ILT-101: 83.3% *vs* placebo: 51.7%; *P* =0.02). ILT-101 was tolerated without systemic side effects and there was no detection of antidrug antibodies. Robust and sustained increases in the numbers and frequencies of the highly suppressive CD4+FoxP3+CD127^lo^CD25^hi^ Treg subset were evident in the IL-2 group. Assessing the characteristics of control groups and the changes of Treg cells could provide a more comprehensive understanding of the observed therapeutic effects. To compare the Treg responses between clinical responders and non-responders of the ILT-101 group, a significant increase in Treg numbers was detectable in the responding patients, suggesting that the clinical outcome is associated with the increased Treg response (120). These results provide strong support for further testing of lower-dose IL-2 therapy in patients with SLE.

**Table 4:** Summary of clinical trials with low-dose IL-2 therapy in systemic lupus erythematosus.

Study type/treatment groups	IL-2 therapy (dose)	Outcomes measured and results	Reference
A double-blind, randomized, placebo-controlled trial in active SLE. IL-2 + SOC (n=50). placebo + SOC (n=50).	1.5 million IU/day of subcutaneous IL-2 (ILT-101) for 5 days followed by 1.5 million IU/week injections for 12 weeks.	The primary end point: SRI-4 response at week 12 (ILT-101: 68%, placebo: 58%; p=0.34), due to a 100% SRI-4 response rate in the placebo group from the two sites. A *post hoc* per-protocol analysis excluded patients from these two sites (n=27): SRI-4 response rate (ILT-101: 83.3%; placebo: 51.7%; p=0.02), SLEDAI and steroid reduction in the IL-2 group, accompanied by the CD4^+^Tregs response. ILT-101 was well tolerated and there was no generation of anti-drug antibodies.	(120)
A double-blind, randomized, placebo-controlled trial in mild to moderate SLE. IL-2 + SOC (n=112). placebo + SOC (n=36).	Pegylated IL-2 (NKTR-358) at 3.0 ug/kg, 6.0 ug/kg, 12.0 ug/kg, 24.0 ug/kg once every 2 weeks, three times in total 4 weeks.	SLEDAI changes are not reported. No SAEs. Increases in numbers and percentages of total and CD25^hi^ Treg were observed. No significant changes in conventional T cells.	(121)
A double-blind, randomized, placebo-controlled trial in active SLE. IL-2 + SOC (n=30). placebo + SOC (n=30).	1 million IU every other day for 2 weeks/cycle for 3 cycles. (14 days between cycles)	Primary end point: SRI-4 response at week 24 (IL-2: 65.5%, placebo: 36.7%; p=0.03), higher lupus nephritis remission rate, increase complement, reduction in anti-DNA antibodies in the IL-2 group. No SAE, increase in Treg cells in the IL-2 group.	(118)
An open-label controlled trial in lupus nephritis. IL-2 + SOC (n=18). SOC (n=12).	1 million IU every other day for 2 weeks/cycle for 3 cycles. (14 days between cycles)	Higher remission rate in the IL-2 group at 10 weeks, improved renal outcomes. No SAE. Increase in Treg cells during cycles.	(122)
An uncontrolled clinical trial in refractory SLE. IL-2 + rapamycin (n=50).	100 WU 3-5 days/month + rapamycin 0.5 mg every other day for 24 weeks	Improvement in SLEDAI after 6,12 and 24 weeks. No SAE Increase in Treg cells during cycles. Reduced Th17 cell/Treg-cell ratio.	(123)
An uncontrolled phase 1/2 clinical trial in refractory SLE. IL-2 + SOC (n=12)	0.75-3.0 million IU/day for 5 days/cycle for 4 cycles (9-16 days between cycles)	Improvement in SLEDAI on day 62, increase in complement, reduction in anti-DNA antibodies. No SAE. Increase in circulating Treg cells and reduction in circulating B cells.	(124)

SOC, standard of care treatment; SRI-4, SLE Responder Index-4; SAE, serious adverse event.

There are new modified forms of IL-2 which exhibit an extended half-life or a higher affinity for trimeric IL-2R in clinical trials (125). IL-2 mutein NKTR-358 with an extended half-life between 7 and 13 days had clinical effects on skin lupus and increased the number and percentage of CD25^hi^ Tregs in a randomized, placebo-controlled phase 1 trial (121). Low-dose IL-2 therapy has been associated with improved lupus disease activity, reduced autoantibody production, and the steroid-sparing effect. The ongoing research is focuses on further understanding the mechanism of low-dose IL-2 therapy, optimizing dosing protocols, and identifying specific patient subgroups that may benefit the most from this treatment approach. Furthermore, long-term safety and efficacy data from large-scale clinical trials are needed to establish the role of low-dose IL-2 therapy as an alternative regimen for SLE (118). In summary, clinical studies have consistently demonstrated that low-dose IL-2 therapy in SLE is well tolerated, and capable of increasing suppressive Treg population that appears to provide complementary immunomodulatory effects on the treatment of SLE.

## 4 Concluding remarks

Elucidation of the roles of various subsets of Treg dedicated to immune homeostasis and the restoration of the balance between Tregs and autoreactive T cells will provide a novel therapeutic potential that governs immune tolerance for the remission of SLE. Although more data on the manipulation and propagation of Tregs subsets and their therapeutic application are still needed, promising clinical responses were observed in patients with SLE who received low-dose IL-2 therapy to balancing Th17 versus Tregs. Future research is warranted to decipher new immunotherapeutics of the balance between Th1/Th17 overactivation and the development of nTregs and peripheral Tregs in SLE.

## Author contributions

Y-GT: Conceptualization, literature search, original draft preparation, visualization, editing. P-FL and H-MW: critical review, editing and commentary. K-HH: critical review, editing, commentary and funding acquisition. C-YL: supervision, critical review, editing commentary and funding acquisition. KY: supervision, conceptualization, critical review, editing, commentary, and funding acquisition. All authors contributed to the article and approved the submitted version.
